# Recent Developments in Antibacterial Therapy: Focus on Stimuli-Responsive Drug-Delivery Systems and Therapeutic Nanoparticles

**DOI:** 10.3390/molecules24101991

**Published:** 2019-05-24

**Authors:** Roberto Canaparo, Federica Foglietta, Francesca Giuntini, Carlo Della Pepa, Franco Dosio, Loredana Serpe

**Affiliations:** 1 Department of Drug Science and Technology, University of Torino, 10125 Torino, Italy; federica.foglietta@unito.it (F.F.); carlo.dellapepa@unito.it (C.D.P.); franco.dosio@unito.it (F.D.); loredana.serpe@unito.it (L.S.); 2 School of Pharmacy and Biomolecular Sciences, Liverpool John Moores University, Liverpool L3 2AJ, UK; f.giuntini@ljmu.ac.uk

**Keywords:** nanoparticles, drug delivery systems, antibiotics drug delivery systems, stimuli-responsive nanoparticles, stimuli-responsive antibiotic-loaded nanoparticles, nanoantibiotics, stimuli-responsive nanoantibiotics, multi-drug resistant bacteria, bacterial biofilms

## Abstract

Conventional drugs used for antibacterial therapy display several limitations. This is not due to antibiotics being ineffective, but rather due to their low bioavailability, limited penetration to sites of infection and the rise of drug-resistant bacteria. Although new delivery systems (e.g., nanoparticles) that are loaded with antibacterial drugs have been designed to overcome these limitations, therapeutic efficacy does not seem to have improved. Against this backdrop, stimuli-responsive antibiotic-loaded nanoparticles and materials with antimicrobial properties (nanoantibiotics) present the ability to enhance therapeutic efficacy, while also reducing drug resistance and side effects. These stimuli can either be exogenous (e.g., light, ultrasound) or endogenous (e.g., pH, variation in redox gradient, enzymes). This promising therapeutic approach relies on advances in materials science and increased knowledge of microorganism growth and biofilm formation. This review provides an overview in the field of antibacterial drug-delivery systems and nanoantibiotics that benefit from a response to specific triggers, and also presents a number of future prospects.

## 1. Introduction

Antimicrobial resistance (AMR) is now considered an emergent global disease and a major public health problem. Epidemiological data from the World Health Organization (WHO) report that there has been an increase in AMR rates in recent years and that this worrying trend is not limited to specific pathogens or to a specific geographical area [1]. AMR is now the second-leading cause of deaths worldwide and the third in the USA, meaning that new strategies to either enhance the effectiveness of existing antibiotics or for the development of new antibiotics are very much needed if this public health emergency is to be addressed by counteracting antibiotic resistance.

Nanoparticle drug-delivery systems are an attractive tool for the treatment of infections. This is thanks to their significant and unique features, such as the improved solubility of hydrophobic antibiotics, prolonged systemic-circulation time and antibiotic half-life, and sustained antibiotic release, all of which may be able to reduce systemic side effects and allow lower drug doses to be administered [2]. A number of improved drug carriers have been developed for the treatment of intracellular pathogens, including antibiotics that are loaded into liposomes and other lipid nanoformulations, silica nanoparticles, microspheres, polymeric carriers, fullerenes, dendrimers and nanoplexes [3,4].

The first generation of drug carriers that was approved for such applications (e.g., Albecet; Sigma-Tau Pharmaceuticals-Ambisome; Astellas Pharma-Amphotec; Ben Venue Laboratories-Arikace; Transave) was primarily based on liposomes, which are spherical vesicles with a lipid bilayer membrane structure that can encapsulate both hydrophilic and hydrophobic agents, thus protecting the cargo (for example small-molecule drugs, nucleotides, proteins, imaging agents or radionucleotides) during its circulation throughout the body [5,6]. They can also be functionalised, for example, with ligands to cell-surface receptors to promote targeting to specific cells and tissues. In addition, they can be coated with polymers to prolong circulation half-life [7,8]. However, although the advantages of liposomes as a drug-delivery system are widely recognised, they also possess some disadvantages for antimicrobial treatment purposes. These include their short shelf-life and stability, encapsulation efficacy and their rapid clearance from the blood stream by phagocytic cells of the mononuclear phagocyte system (MPS), which is also referred to as the reticuloendothelial system (RES) [9]. In addition, the design of an optimal drug carrier for the release of antibiotics must always take into consideration the most important points in antibiotic treatment: (i) improving antimicrobial-treatment efficacy; (ii) increasing local drug concentration at the site of the infection; (iii) minimising antibiotic accumulation in healthy host tissue; (iv) reducing the risks of toxicity and the exposure of commensal microflora to sub-lethal doses of antibiotics, which can promote the development of antimicrobial resistance [10]. The design of stimuli-responsive systems that either recognise the bacterial microenvironment and react in a dynamic way, or that are susceptible to specific physical stimulation, is therefore one of the possible ways to improve antibiotic drug-delivery systems and enhance their targeting properties, efficiency and the efficacy of antibiotic therapies while, at the same time, minimising side effects and developing new strategies against AMR.

**Figure 1:**
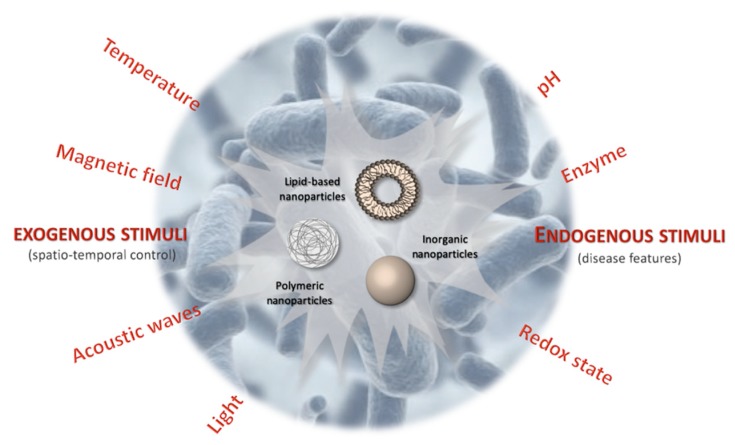
Schematic illustration of stimuli-responsive antibiotic drug-delivery systems. Lipid-based, polymeric and inorganic nanoparticles are the most studied stimuli-responsive drug delivery systems for antibiotic drugs.

The idea of stimuli-responsive systems was first introduced in the late 1970s with the use of thermosensitive liposomes for the local release of drugs through hyperthermia [11]. Since then, a vast body of research on stimuli-responsive materials, and especially on their design and applications, has been accumulated [12]. Nanoscale stimuli-responsive devices can be sensitive to specific intracellular stimuli, such as pH, the varying concentrations of certain enzymes or chemical compounds (e.g., glutathione). They can also be sensitive, on the tissue level, to specific microenvironmental changes that are associated with pathological conditions such as inflammatory disease, ischemia and, of course, infections. Furthermore, sustained drug release and improvements in inherent therapeutic nanomaterials can be achieved by extracorporeal physical stimuli, such as magnetic-, thermal-, light- and ultrasound (US)-sensitive nanoparticle systems. In this regard, stimuli-responsive drug-delivery systems can provide antibiotic drug-delivery systems with a great many advantages (Figure 1), including enhancing the targeting properties, efficiency and efficacy of antibiotic therapies while, at the same time, minimising their side effects. Indeed, the emergence and dissemination of drug-resistant pathogens entails the necessity of decreasing doses while increasing treatment efficacy. The use of external or internal stimuli to influence the antibiotic activity of responsive drug-delivery systems can lead to smart therapeutic responses such as: (i) controlled release of payload in the targeted biological compartment and (ii) rapidly addressing the pathological event. Furthermore, an important feature of this type of approach is the reversibility since the antibiotic nanosystem may return to its initial state upon application of the specific stimulus. In this review, we provide an overview of the current progress in the field of stimuli-responsive drug delivery systems (DDSs) and stimuli-responsive therapeutic nanoparticles for the management of microbial infection and AMR.

## 2. Exogenous Stimuli-Responsive Antibiotic Drug-Delivery Systems

Effort in the development of stimuli-responsive drug-delivery systems has traditionally been focused on the synthesis and modification of biomaterials, such as polymers and lipids, in order to provide them with a wide range of chemical functionalities that readily undergo hydrolysis, enzymatic degradation, or conformational changes in response to either chemical (e.g., pH, ionic strength, reactive oxygen species), physical (e.g., temperature, light, ultrasound ), or biological (e.g., proteolytic enzymes) stimuli and thus induce therapeutic-cargo release. In this regard, the co-formulation of metallic inorganic nanoparticles (NPs) such as Au NPs, Ag NPs, Fe_2_O_3_ NPs and Fe_3_O_4_ NPs with traditional drug-delivery systems, such as liposomes, polymersomes and hydrogels, is a promising and emerging approach to the stimuli-responsive release of antibiotics [13]. This development is underpinned by the rapid advancements that have been made in the synthesis of monodispersed and biocompatible inorganic NPs with a wide range of surface functionalisation and physicochemical properties. The selective release of therapeutic cargo from the drug-delivery systems in response to a variety of remotely applied and local stimuli, such as light irradiation, magnetic fields, and pH, can be achieved by harnessing the unique optical, magnetic, and/or physicochemical properties of metallic inorganic NPs.

### 2.1. Thermal Release

The unique optical and magnetic properties of inorganic NPs have been harnessed for the photothermal- and magnetothermal-mediated release of therapeutics from drug delivery systems. This is thanks to the ease of achieving selective drug-carrier disruption via the external application of a laser or alternating magnetic field. The generation of heat from activated inorganic NPs that are incorporated into drug-delivery systems also enhances the antibacterial efficiency of the delivered antibiotics through synergistic thermal-ablative effects [14].

#### 2.1.1. Photothermal Release

Photothermal heating occurs due to the absorption of light by an ensemble of electrons on the surface of certain conductive materials and the subsequent dissipation of that energy as heat. Plasmonically active metal NPs, such as Au NPs, Ag NPs, and Cu NPs, are particularly effective photothermal agents because of their large absorbance cross sections, tunable optical properties, and highly efficient conversion of light into heat [15]. In such metallic NPs, most of the energy gained by the electrons during photo-excitation at this resonance is dissipated as heat, through electron-photon collisions, leading to the heating of the NPs and the surrounding environment. Photothermal heating can either be used to deliver thermal energy to a localised area as reported by Khan and colleagues in their research work [16], or, alternatively, throughout the drug carrier, thereby causing the destruction of the encapsulating material and triggering the release of the loaded therapeutics. Some early demonstrations of the use of photothermal effects to release antibacterial agents from drug carriers have been reported in a number of research studies [17,18,19,20].

In Radt et al. [17], lysozyme crystal and Au NPs were embedded into a polyelectrolyte shell built from the layer-by-layer deposition of negatively charged poly(sodium 4-styrenesulfonate) and positively charged poly(allylamine hydrochloride). The capsules were first irradiated with a 10 ns pulsed laser (λ = 1064 nm) at 50 mJ cm^−2^ for 5 min at a frequency of 10 Hz before being added to a suspension of *Micrococcus lysodeikticus*. The release of lysozymes from photothermally degraded capsules led to rapid bacterial digestion being observed. In the work by Amoli-Diva and co-workers [18], Ag NPs were embedded within a poly(butyl methacrylate-co-acrylamide-co-methacrylic acid) hydrogel to provide the controlled release of the model antibiotic ofloxacin following irradiation with a 405 nm laser for 15 s at the 10, 30 and 50 min after the start of the experiment. After 70 min, a significantly higher amount of ofloxacin (>80% of total mass) was released from these samples than from the non-irradiated samples.

In a further study, Liu et al. [19], developed an efficient antimicrobial hybrid for combined chemo-photothermal therapy that is based on polydopamine (PDA)-coated gold nanorods (GNRs). The PDA coating achieved high silver-ion loading efficiency and glycol chitosan (GCS) functionalisation (Ag^+^-GCS-PDA@GNRs). The authors tested their bactericide hybrid on in vitro co-cultured drug-resistant Gram-positive methicillin-resistant *Staphylococcus aureus* (MRSA) or Gram-negative *Escherichia coli*. Furthermore, photothermal therapy was introduced to construct a chemo-photothermal combinational bactericide system and further improve the antibacterial effect. The test samples were therefore further irradiated with an 808 nm-laser at 0.5 W cm^−2^ for 7 min. The bacterial inactivation percentages for the Ag^+^-GCS-PDA@GNRs + near-infrared (NIR) treatment group were 99.6 and 96.8% for MRSA and *Escherichia coli*, respectively. In addition, in vivo biocompatibility and in vivo antibacterial activity was also investigated using a murine subcutaneous abscess model. In the animal group where Ag^+^-GCS-PDA@GNRs were intravenously injected and further exposed to 808 nm NIR irradiation for 6 min at 0.8 W cm^−2^, the mice showed no evident inflammation and abscess. In conclusion, the authors suggest that this antimicrobial depot will be able to circulate freely in the bloodstream and accumulate in acidic infectious sites where they become positively charged, and selectively target negatively-charged bacteria-cell surfaces. With external light-activation, they will generate heat in the close vicinity of the target bacteria. The acidity/thermally-triggered Ag^+^ ion release may reduce the heat resistance of the bacteria and photothermal therapy may promote the antibacterial efficiency of the Ag^+^ ions.

In the work by Borzenkov and colleagues [20], poly(vinyl alcohol) (PVA) films containing five-branched gold nanostars were developed and displayed a pronounced photothermal effect upon irradiation at three NIR laser wavelengths (λ = 730, 800 and 1064 nm). It was shown that the local NIR-induced increase of temperature was sufficient to eradicate *Escherichia coli* grown on the film surface.

#### 2.1.2. Ultrasound Release

Thermal release has also been exploited for the mechanical disruption of drug carriers via US exposure [21,22]. In the study by Wu et al. [23], the pulsed-laser irradiation of liposomes that either encapsulated hollow gold nanoshells (HGNs) or that were mixed with free HGNs triggered the release of encapsulated 6-carboxyfluorescein (CF), a fluorescent dye, above a threshold of ≈1.5 W cm^−2^ without the induction of permanent liposomal damage or drastic temperature increases to the bulk solution. This suggests that triggered CF release occurred due to transient cavitation effects. The authors suggested that the water molecules surrounding the HGN vaporise at sufficiently high temperatures to form a gaseous cavity that violently expands and collapses. This is analogous to the transient cavitation effects of microbubbles in response to applied US. The collapse of the vapour bubble subsequently emits a shock wave that propagates through the surrounding medium to transiently disrupt liposome membranes and thus release the encapsulated cargo.

In this regard, US appears to be paying an ever-increasing role in the delivery of therapeutic agents, including genetic material, proteins and chemotherapeutic agents, such as antibiotics, via micro- or nano-bubble cavitation, without the involvement of bulk heating [24]. Cavitation is the formation and/or activity of gas-filled bubbles in a medium that is exposed to US. As the pressure wave passes through the media, gas bubbles of any size will expand at low pressure and contract at high pressure. If the resulting oscillation in bubble size is fairly stable (repeatable over many cycles), the cavitation is called “stable” or “non-inertial” cavitation. This type of oscillation creates a circulating fluid flow (called microstreaming) around the bubble, with velocities and shear rates that are proportional to the amplitude of the oscillation. At high amplitudes, the associated shear forces are capable of shearing open red-blood cells and synthetic vesicles, such as liposomes [25]. Cavitation therefore, appears to play two roles: it disrupts the structure of the carrier vesicle and releases the drug, but it also makes cell membranes and capillaries more permeable to drugs. In a study by Dong et al. [26], microbubbles were injected subcutaneously, in order to increase US cavitation, into an in vivo rabbit model into which two *Staphylococcus epidermidis* infected catheters had previously been implanted. The animal group that was treated with vancomycin, US (300 kHz, 0.5 W/cm^−2^, 50% duty cycle) and microbubbles was compared to the control group and other animal groups. The antibacterial activity of vancomycin against *Staphylococcus epidermidis* biofilms was found to be enhanced by three orders of magnitude, compared to the control, confirming that US cavitation can play an important role against infections, especially against these communities of microorganisms that can attach to both abiotic and biotic surfaces [27]. Biofilms consist of microorganisms with altered phenotypes that live in a self-organised, cooperative community that is attached to surfaces and each other and embedded into a self-produced matrix of exopolymer saccharides. Biofilms are relevant for home care and hospice clinicians because the colonisation of medical devices plays a key role in the issue of healthcare-associated infections [28].

In another work [29], the authors showed that the focused US and gentamicin-encapsulated liposomes reduced the number of viable bacteria in alginate-based *Ralstonia insidiosa* biofilms by approximately 72%, compared to US alone, gentamicin in solution alone (no liposomes) and gentamicin in solution with high-intensity focused US. These data show that high-intensity focused US in combination with an antibiotic strategy can kill bacteria within synthetic alginate biofilms, while non-focused US was able to increase the attachment of liposomes to the biofilm, as demonstrated by the same authors [30].

#### 2.1.3. Magnetothermal Release

The magnetothermally stimulated release of antibiotics from drug-delivery vehicles has been demonstrated using superparamagnetic iron oxide nanoparticles (SPIONs) [31,32,33,34]. SPIONs consist of cores made of iron oxides that can be targeted to the required area using external magnets. SPIONs exhibit a range of favourable properties, including intrinsic magnetism for MRI, safety and the availability of biocompatible coatings and surface functional moieties. These features opened up a wide range of possible applications for SPIONs in drug delivery [35], in vivo medical imaging [36], biosensing [37], regenerative medicine [38] and hyperthermia [39]. SPION-based DDSs are commonly composed of magnetite and maghemite NPs with an organic or inorganic coating. These magnetic drug-bearing nanostructures rely on external magnetic-field guidance to reach their target tissue. Magnetic vehicles, including magnetic capsules, magnetoliposomes and magnetodendrimers reduce the clearance of drugs and increase their blood-circulation time. They also increase drug-internalisation efficiency in target cells and minimise nonspecific cellular interactions, thus reducing the total required dose and associated side effects. Their unique superparamagnetic characteristics mean that SPIONs become magnetised up to their saturation magnetisation, using an external magnetic field, and gain high magnetic susceptibility. No magnetic interaction is observed upon the removal of the magnetic field. In this way, SPIONs acquire the capability to transport the encapsulated or attached drug to the target site in the body under an applied magnetic field and are inactivated upon the removal of the magnetic field [40].

In this regard, Mohapatra and colleagues used chitosan-based microbeads that were loaded with magnetite SPIONs to demonstrate the on-demand release of vancomycin [34]. In this system, the SPIONs were incorporated during the microbead formation, which involves the cross-linking of chitosan and vancomycin to poly(ethylene glycol) dimethacrylate (PEGDMA). Following the exposure of the SPION-loaded microbeads to a 25 mT alternating magnetic field (AMF) at 109.9 kHz for 30 min, the generation of localised magnetothermal heating, three, five and seven hours from the start of the experiment, provided sufficient energy to cleave the ester bonds linking the vancomycin to the microbeads. This resulted in a significantly higher amount of vancomycin being released from the microbeads, to levels above the minimum inhibitory concentration (MIC), than those that were released by the non-stimulated control, which remained below the MIC. To further confirm the suitability of this approach to the antimicrobial field, Sirivisoot and co-workers [32], presented the release of ciprofloxacin, which was encapsulated within polycaprolactone-iron-oxide NPs after exposure to an alternating magnetic field for 1 day, in a *Staphylococcus aureus* broth inhibition assay. The authors also pointed out that the magnetothermal response of a SPION may differ from one iron oxide form to another. Indeed, the co-loading of either maghemite or hematite NPs with ciprofloxacin in polycaprolactone microspheres exhibited differences in the release and also in the bactericidal efficacy of ciprofloxacin against *Staphylococcus aureus* after exposure to an alternating magnetic field. Higher release and efficacy was observed in the maghemite-loaded microspheres than in the hematite-loaded microspheres.

### 2.2. Magnetic Release

The superparamagnetic properties of SPION might also be an ideal way to promote the release, upon the use of an external magnetic field, of antibiotics in unique bacteria microenvironments, such as a biofilm, where highly dynamic communities of immobile bacteria and a protective extracellular polymeric matrix provide the conditions for the development of innate resistance to both antimicrobial compounds and host-response factors. Geilich et al. [41], demonstrated the biofilm-eradication effectiveness of this method using iron oxide polymersome (IOPs) formed from methoxy poly(ethylene glycol)-*b*-poly(d,l-lactic acid) diblock co-polymers to co-encapsulate methicillin within the aqueous core and hydrophobic SPIONs within the membrane bilayer. In this work, biofilms formed from *Staphylococcus epidermidis* were grown on glass cover slips and incubated with IOPs containing 20–100 μg mL^−1^ of SPIONs and 10–50 μg mL^−1^ methicillin for 24 h. A magnetic field was subsequently used to pull the IOPs through the biofilm. The application of the IOPs and the magnetic field to the biofilm resulted in the effective penetration of the IOPs through the biofilm and a selective bactericidal effect within the region of the applied magnetic field. Importantly, complete biofilm eradication was observed using IOPs that contained SPIONs and methicillin at a lower concentration than the minimum bactericidal concentration (MBC) value of free methicillin for *Staphylococcus epidermidis* grown within a biofilm.

## 3. Endogenous Stimuli-Responsive Antibiotic Drug-Delivery Systems

pH variation has been exploited to control the delivery of drugs to specific organs (i.e., the gastrointestinal tract or the vagina) and intracellular compartments (such as endosomes or lysosomes), and to trigger the release of the drug when subtle environmental changes are associated with pathological situations, such as cancer or infection. Two main strategies exist: (i) the use of polymers (polyacids or polybases) with ionisable groups that undergo conformational and/or solubility changes in response to environmental pH variation; (ii) the design of polymeric systems with acid-sensitive bonds whose cleavage enables the release of molecules that are anchored to the polymer backbone [12].

In this regard, Radovic-Moreno and colleagues [42], developed vancomycin-encapsulated, pH-responsive, surface charge-switching poly(d,l-lactic-co-glycolic acid)-*b*-poly(l-histidine)-*b*-poly(ethylene glycol) nanoparticles for the treatment of *Staphylococcus aureus* infection. The results suggest that the promotion of NP-bacteria interactions under acidic conditions can partially mitigate the loss of vancomycin activity with pH, which highlights the potential of this drug-delivery system for the treatment of infections that are associated with localised acidity. Furthermore, pH responsiveness has also been used in the treatment of resistant intracellular infections as bacteria are located in acidic intracellular compartments. Sémiramoth and co-workers [43], described innovative nanoparticles that are based on the bioconjugation of penicillin G (PNG) to squalene (SqPNG) and that are designed to release penicillin G within acidic subcellular organelles (SqPNG-pH), such as lysosomes, in order to overcome severe intracellular infections by pathogens whose mechanisms of resistance arise from the poor intracellular diffusion of a range of antibiotics. The antibacterial activity of SqPNG-pH NPs was investigated against *Staphylococcus aureus*, which is able to invade and survive in macrophages. The data clearly show that SqPNG-pH NPs present a significant ability to kill intracellular *Staphylococcus aureus* (87% inactivation) compared to with all the other treatments, i.e., free PNG or SqPNG NPs. Kalhapure and colleagues [44], have recently synthesised a new lipid with a cleavable acetal linkage as part of their development of pH-responsive solid lipid nanoparticles (SLNs) that can more specifically deliver vancomycin in an acidic environment to control the growth of methicillin-sensitive *Staphylococcus aureus* (MSSA) and MRSA.

Hydrogels are a class of highly hydrated biomaterials that are usually produced from natural or synthetic polymers. Many hydrogels are biocompatible and can be designed to have mechanical properties similar to natural tissues, and thus have been used in a myriad of applications including drug delivery [45]. Their hydrophilic nature means that some antibiotics, such as ciprofloxacin [46,47,48] and vancomycin [49], have been used to prepare active gels. Although these carriers were able to provide the controlled release of antimicrobial agents and had good antibacterial activity, the release profiles from these carriers were found to be load-dependent and time-dependent and to lack the ability to respond smartly to the microenvironment of the bacterial infection. pH-responsive antibiotic delivery systems have therefore gained considerable attention thanks to their enormous potential and ease of application in the clinical treatment of infections, such as the eradication of *Helicobacter pylori*, for which antibiotics need to penetrate the gastric mucus layer and maintain a concentration sufficient for antibacterial activity in the infected site for a suitable length of time. In order to tackle this issue, Chang et al. [50], developed a pH-sensitive hydrogel that incorporate chitosan/γPGA NPs that are loaded with amoxicillin. They investigated the physicochemical characteristics of the hydrogels and nanoparticles as a function of pH values in a human gastric adenocarcinoma cell line and at the site of *Helicobacter pylori* infection. They found that the system protects the drug from destruction by gastric acid, and that the amoxicillin interacted specifically with the intercellular spaces and with the cell cytoplasm of the site of *Helicobacter pylori* infection. In a broader antimicrobial context, pH-responsive hydrogels may well be used as controlled-release systems to treat a range of different infections. Lu and colleagues [51], designed a pH-sensitive poly(acrylic acid) (PAA) and *p*-(2-(dimethylamino)ethyl methacrylate) (PDMAEMA) composite hydrogel. The hydrogel decomposed gradually when placed in a weak acidic microenvironment, but was stable under normal physiological and weakly basic environments. The authors investigated the in vitro release rates of vancomycin and levofloxacin from the hydrogel using a UV spectrophotometer at a number of pH values (5.5, 7.3 and 9.1). Both drugs were completely released at pH 5.5 due to the degradation of the hydrogel under these acidic conditions. The antibacterial activity of vancomycin-loaded hydrogel provided decreased *Staphylococcus aureus* proliferation activity in 4 h, and 99% of the bacteria was killed after 6 h. A similar approach was proposed by Anirudhan et al. [52]. In their work, gentamicin (GS), ampicillin (Amp) and 2-amino guanidine, which was used to limit the side effects of GS, were encapsulated in a pH sensitive gelatin methacrylate/methacrylic acid hydrogel. Swelling and drug-release studies confirms that the external pH was the triggering force for the release of the drugs, while in vitro antimicrobial studies against *Escherichia coli* and *Staphylococcus aureus* confirmed the increased antibacterial effect of the drug combination.

pH-sensitive liposomes have been extensively used as an alternative to conventional liposomes for the intracellular delivery of therapeutics/antigen/DNA/diagnostics to various target-cell compartments [53]. A number of researchers have suggested a range of strategies for the use of pH-sensitive liposomes in the antimicrobial field. Lutwyche and colleagues [54], encapsulated gentamicin in a pH-sensitive liposomal formulation and characterised its delivery to *Salmonella typhimurium* and *Listeria monocytogenes*, which resided in cultured macrophages. More recently, the same authors [55], investigated the same formulation in vivo in a mouse model that bore a systemic *Salmonella enterica* serovar *typhimurium* infection. The study showed that the encapsulation of gentamicin in pH-sensitive liposomes significantly increased the concentrations of the drug in plasma, the liver and spleen, compared to those of free gentamicin in the murine salmonellosis model, and that the antibacterial activity was 10^4^-fold higher than for free gentamicin. A similar approach, but against a basidiomycetous yeast that causes serious infections of the lungs and central nervous system in immunocompromised patients, was introduced by Nasti et al. [56]. The authors evaluated the efficacy of pH-sensitive liposomes of nystatin against *Cryptococcus neoformans* infection in a murine model. They found that the use of pH-sensitive liposomes significantly enhanced the intracellular delivery of nystatin, and showed enhanced antifungal activity, in terms of increased survival rate, while fungal burden in brain and liver was reduced. Furthermore, significant nystatin toxic effects, which were observed in the free form, were avoided. As previously described, although liposomes have been extensively studied as antimicrobial delivery vehicles and present proven advantageous features (i.e., high biocompatibility, unique bilayer structure that can fuse with bacterial membranes, high drug-carrying capacity and readily tunable formulation properties), their applications are often limited by poor stability, which is caused by spontaneous fusion among liposomes, leading to payload loss and non-reproducible mixing. Several attempts have been made to overcome these drawbacks by coating their surface with a stealth material such as polyethylene glycol (PEG) [57,58], and zwitterionic polymers [59], but these liposomes are rarely used for antimicrobial delivery to treat bacterial infections. This is mainly due to the fact that the polymer coating not only stabilises the liposomes against fusion with each other, but also prevents them from fusing with bacterial membranes across which the antimicrobial payload must be delivered. The engineering of liposomal formulations that are stabilised against fusion prior to arriving at target bacteria, while maintaining their ability to fuse with cell membranes at the infection sites would be the ideal situation. In this regard, Thamphiwatana and co-workers [60], have developed negatively charged doxycycline-loaded liposomes that are electrostatically stabilised with small cationic chitosan-coated Au NPs for the gastric delivery of antibiotics to treat *Helicobacter pylori* infections. The authors demonstrated that, at acidic pH, the chitosan coating on the Au NPs is predominantly protonated, thus enabling strong binding to the liposomes and promoting their stabilisation via electrostatic repulsions between the Au NP-coated liposomes. However, the deprotonation of the chitosan coating on the Au NPs occurs at neutral pH, leading to the detachment of the Au NPs from the liposomes and to the latters’ subsequent fusion with a bacterial membrane, which releases the encapsulated doxycycline to eradicate the bacteria. Indeed, the authors demonstrated that their system had superior antibacterial efficacy against *Helicobacter pylori* than the same concentrations of free doxycycline.

Finally for this field, micelles have demonstrated several attractive advantages over other types of carriers; higher stability than liposomes and stronger responsiveness to stimuli than nanoparticles. Indeed, the hydrophilic outer corona of a micelle is able to reduce the uptake by the reticuloendothelial system, prolonging in vivo circulation, whereas their hydrophobic inner core is beneficial for the high-capacity loading of poorly water-soluble chemical compounds, such as antibiotics. Chen et al. [61], therefore developed a novel pH and lipase-sensitive vancomycin (Van)-hydrazone-poly(ethylene glycol)-poly(ε-caprolactone)/ciprofloxacin (Cip) micelles (Van-hyd-PECL/Cip) to achieve bacterial targeting using an antibiotic (Van) and the on-demand release of antibiotics (Van and Cip) to combat bacterial infections. Their results included the higher survival of *Pseudomonas aeruginosa*-infected mice, lower bacterial burden and decreased alveolar injuries in the lungs, compared with micelles used without the inoculation of Van moieties, and free drugs. Triple doses of Van-hyd-PECL/Cip micelles further extend animal survival, decrease the bacterial colonisation of the lungs and almost completely restored normal alveolar microstructure.

## 4. Antibiotic Nanoparticles

**Figure 2:**
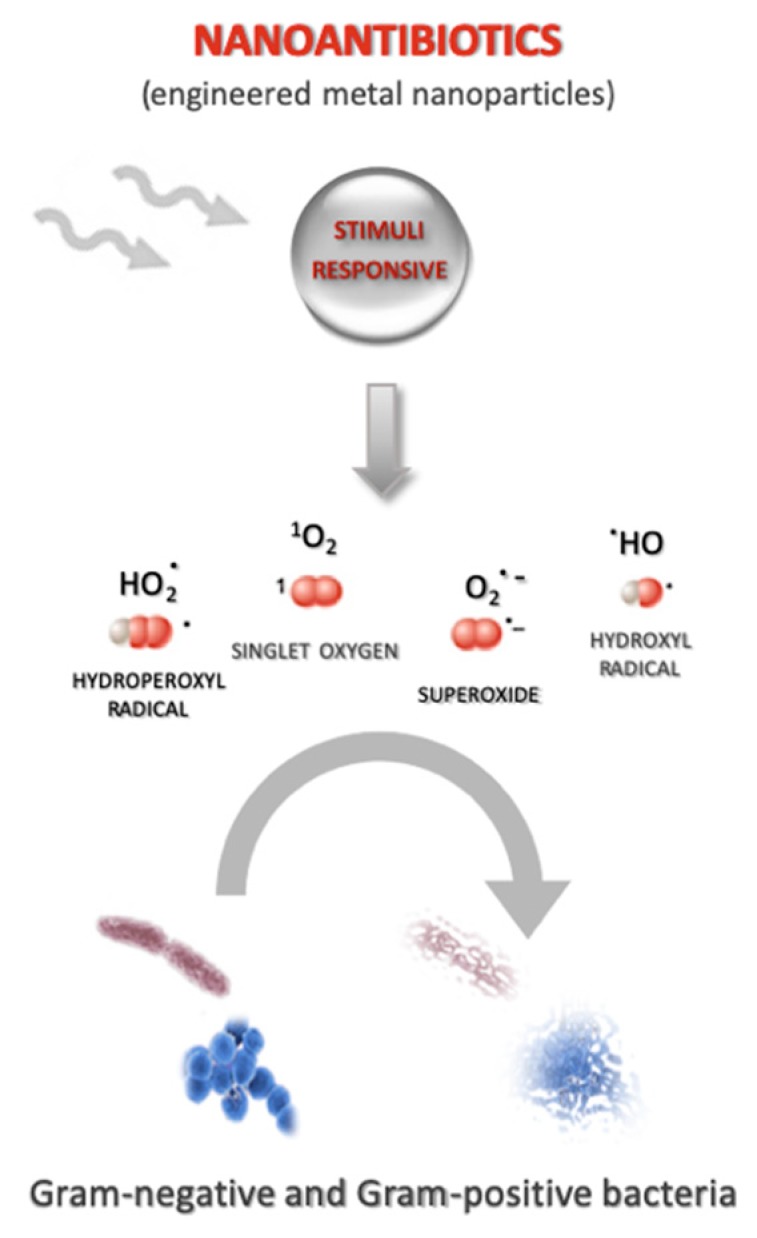
Schematic illustration of nanoantibiotics. One of the proposed antimicrobial mechanisms of stimuli-responsive nanontibiotics (nAbts) is the generation of reactive oxygen species (ROS) such as singlet oxygen, hydroxyl radicals, superoxide and hydroperoxyl radicals that can affect either Gram-negative and Gram-positive bacteria.

Antibiotic drug-delivery systems and antibiotic stimuli-response drug-delivery systems, as previously described, offer undoubted advantages, in terms of resistance and side effects, compared to conventional antibiotics. However, translation to the clinic is still beyond reach for most approaches. Only liposomes and lipid nanoparticles for amphotericin delivery, including Abelcet^®^, Amphotec^®^, Ambisome^®^, and Arikayce^®^, are currently approved for use in patients. The identification of novel and efficient antimicrobial therapies therefore still remains a priority in the war against infections. The use of nanoantibiotics (nAbts) as a therapeutic strategy has recently gained a lot of attention [62,63]. nAtbs are nanomaterials that have antimicrobial activity or improve the efficacy and safety of antibiotic administration [64]. nAbts have many advantages over conventional antibiotics, including production, storage, durability and versatility, while preparation may also be cheaper, faster and more adaptable, with the added advantage of a long shelf-life. Compared to conventional antibiotic drug-delivery systems, nAbts show higher surface-area-to-volume ratios and unique chemical-physical properties. They are typically composed of either naturally occurring antibacterial substances, metals and metal oxides, carbon-based nanomaterials, or nanoemulsions. Some authors have proposed that the antimicrobial mechanisms of nAbts include the generation of reactive oxygen species (ROS) as reported by Dai and colleagues in their research work [65] (Figure 2), the disruption/compromising of the bacterial cell wall/membrane, the interruption of energy transduction and the inhibition of enzyme activity and DNA synthesis [66]. Although some mechanisms appear to overlap with those of conventional antibiotics, the actual mechanisms by which these occur are vastly different; nAbts physically disrupt key biological process, while conventional antibiotics interfere on the molecular scale.

nAtbs come in a range of chemical compositions, shapes and sizes, and their chemical-physical versatility clearly indicates that there is a synergistic opportunity to be taken in designing nAbts with a stimuli-responsive linker [66], such as those for temperature, light, pH, redox and enzymes. In this regard, interesting are the antimicrobial strategies called “photoantimicrobial chemotherapy” (PACT) and “sonoantimicrobial chemotherapy” (SACT) [67]. These approaches, similar to photo- and sonodynamic therapy [68,69], rely on two individually harmless components, i.e., a sensitiser and a physical trigger as light or US. The irradiation of the sensitiser with light or US initiates a chain of events that culminates with the production of highly reactive cytotoxic species, which rapidly lead to bacteria death. The combination of photoirradiation and sensitiser exposure has been reported as a potential bactericidal approach. In a work by Dhal et al., the authors used a combination of photodynamic exposure and rose bengal on several Gram-positive species (*Bacillus subtilis*, *Staphylococcus aureus*, *Streptococcus faecalis* and *Streptococcus salivarius*) and Gram-negative *Salmonella typhimurium*. They observed a 99% inactivation of Gram-positive bacterial species, as compared to *Salmonella typhimurium*. This led to the hypothesis the mechanism of rose bengal anion penetration through the outer portion of the Gram-negative cell wall to a critical location within the cell for effective photosensitisation is effectively different [70]. Recently, a study by Sabbahi and colleagues reported the antimicrobial activity of dianionic rose bengal disodium salt, under photodynamic exposure, on *Staphylococcus aureus*. The synergism between photosensiser and light exposure induced a 79.4% reduction in bacterial viability. Specifically, hydroxyl radicals played the most important role in the photoinactivation of the tested bacteria by the dianionic rose bengal [71].

A few examples of the antimicrobial roles of nAtbs under stimuli response will be reported herein (Table 1). Nitric oxide shows some potential antimicrobial activity thanks to its cytotoxic effect, which is associated with radical species production. Specifically, Consoli and colleagues described the antibacterial activity of a calixarene-NO donor conjugate, when exposed to light treatment, towards *Staphylococcus aureus* and *Escherichia coli*, which were chosen as representatives of the Gram-positive and Gram-negative bacteria strains [72]. Using calixarene as cages for the storage and release of gaseous NO, a significant biocidal effect towards *Staphylococcus aureus* count was observed upon visible light irradiation, in an irradiation-time dependent fashion. The same effect was not observed on *Escherichia coli*, and it was thought that this was because of the different way the Gram-negative bacteria are organised. Therefore, an interesting photoresponsive effect was provided by calixarene-NO complex [72].

A further interesting antimicrobial strategy is the use of metallic nanoparticles, such as Ag NPs [73]; whereas synergic activity between silver ions release and hyperthermia caused by the photo-thermal effect under near-infrared laser irradiation occurs [74,75,76]. In this regard in a work by Ballesteros, the authors reported the use of nanogels that were loaded with metallic nanoparticles and presented the synergic activity of this platform after irradiation with a specific wavelength. This irradiation generates local electronic vibrations which lead to changes in polymeric structure, releasing the nanoparticles into the surrounding environment. Silver nanoparticles stand out from other nanoparticles that display antibacterial properties as the silver ions that are released from the crystalline core can produce chemical disequilibrium in bacterial cells. Ballesteros and colleagues demonstrated that the irradiation of a nanogel at 405 nm induces the excitation of Ag NPs and thus the rupture of cross-linking, leading to its release over time. In this context, the kinetics of Ag NP release under irradiation with a 405 nm diode LED was investigated in Gram-negative *Escherichia coli* [77].

A great amount of information, which has been accumulated over the last 20 years, has demonstrated the strong bactericidal activity of titanium dioxide (TiO_2_), which is a commonly used semiconductor photocatalyst, upon irradiation with near-UV light and UVA. The required concentration for bacteria-killing varies over the 100–1000 ppm range, depending on TiO_2_ NP size and the intensity and wavelength of the light, although it has been mainly attributed to the production of free-hydroxyl radicals and peroxide [62]. A new study by Yadav and colleagues has reported that the antibacterial efficiency of Ni-TiO_2_ NPs under light exposure is higher towards Gram-positive *Staphylococcus aureus* and *Bacillus subtilis*, than towards Gram-negative *Escherichia coli* and *Salmonella abony*, which is seemingly determined by the complexity and the density of the cell membrane/wall [78].

## 5. Conclusions

The use of stimuli-responsive nanoparticles shows great promise as it may provide long-term solution to the main drawbacks in antimicrobial therapy: resistance and the severity of side effects. One of the major advantages provided by this approach is the spatial and temporal release of antibiotics from drug carriers at an infection site. This is achieved by photo, magneto, thermal, US and pH stimuli, whereas stimuli-responsive nAbt systems allow traditional antibiotic discovery pathways to be bypassed. Moreover, the therapeutic relevance in this field is confirmed by the role in other fields such as cancer treatment, vaccine development and theranostic applications. In particular, this approach seems better than existing due to the controlled and precise delivery of the antibacterial agents improving the therapeutic effect mainly on abscesses and infected wounds, with a reduction of systemic side-effects. Furthermore inorganic NPs, by external stimuli, can also improve penetration of antibiotics through difficult-to-treat biofilms as seen with SPIONs and Ag NPs. Regarding the manufacturing aspect, surely inorganic NP can be preferred as they can confer stimuli-responsive properties in a simpler and more reproducible manner. However, while promising in vitro antibacterial effects have been observed, these novel systems are still in the relatively early stages of development and certain challenges associated with the use of different types of nAbts and stimuli-responsive drug-delivery systems for the release of antibiotics exist and still need to be overcome. These include technology limitations in providing a stimulus to nAbts or delivery systems. For example, the limited tissue-penetration depth of light can restrict its application to superficial infections even though the near-infrared laser irradiation could overcome this issue due to a greater penetration depth. Furthermore, the need for the co-development of a cost-effective magnetic-field generator that is suitable for use against a range of infection types is an issue in the case of the magnetic-based systems. Crucially, the toxicity of the materials, especially inorganic materials, remains a challenge. However, a systematic method that address these challenges may well pave the way for innovative therapeutic avenues without end.

## Figures and Tables

**Table 1:** Examples of stimuli-responsive nanoantibiotics (nAtbs).

Type	Average NP Diameter	Stimulus	Mechanism of Antibacterial Action	Application	Reference
Calixarene-NO donor conjugate	270 nm	Light (400 nm)	Damage of cell membrane by NO release	*S. aureus*, *E. coli*	[70]
Nanogel of aniline and chitosan-containing Ag NPs	78 nm	Light (405 nm)	Damage of cell membrane by Ag NP release	*E. coli*	[71]
Ni-TiO_2_ NPs	10 nm	Light (400 nm)	Damages of cell membrane by ROS generation	*S. aureus*, *B. subtilis*, *E. coli* and *S. abony*	[72]
